# Quantitative Analysis of Mouse Urine Volatiles: In Search of MHC-Dependent Differences

**DOI:** 10.1371/journal.pone.0000429

**Published:** 2007-05-09

**Authors:** Frank Röck, Karl-Peter Hadeler, Hans-Georg Rammensee, Peter Overath

**Affiliations:** 1 Institut für Physikalische Chemie, Interfakultäres Institut für Zellbiologie, Universität Tübingen, Tübingen, Germany; 2 Biomathematik, Universität Tübingen, Tübingen, Germany; 3 Abteilung Immunologie, Interfakultäres Institut für Zellbiologie, Universität Tübingen, Tübingen, Germany; Sanofi-Aventis, United States of America

## Abstract

Genes of the major histocompatibility complex (MHC), which play a critical role in immune recognition, influence mating preference and other social behaviors in mice. Training experiments using urine scent from mice differing only in the MHC complex, from MHC class I mutants or from knock-out mice lacking functional MHC class I molecules (ß2m-deficient), suggest that these behavioral effects are mediated by differences in MHC-dependent volatile components. In search for the physical basis of these behavioral studies, we have conducted a comparison of urinary volatiles in three sub-strains of C57BL/6 mice, a ß2m-deficient mutant lacking functional MHC class I expression and two unrelated inbred strains, using the technique of sorptive extraction with polydimethylsiloxan and subsequent analysis by gas chromatography/mass spectrometry. We show (i) that qualitative differences occur between different inbred strains but not in mice with the C57BL/6 background, (ii) that the individual variability in abundance in the same mouse strain is strongly component-dependent, (iii) that C57BL/6 sub-strains obtained from different provenance show a higher fraction of quantitative differences than a sub-strain and its ß2m-mutant obtained from the same source and (iv) that comparison of the spectra of ß2m mice and the corresponding wild type reveals no qualitative differences in close to 200 major and minor components and only minimal differences in a few substances from an ensemble of 69 selected for quantitative analysis. Our data suggest that odor is shaped by ontogenetic, environmental and genetic factors, and the gestalt of this scent may identify a mouse on the individual and population level; but, within the limits of the ensemble of components analysed, the results do not support the notion that functional MHC class I molecules influence the urinary volatile composition.

## Introduction

In 1975 Lewis Thomas hypothesized that “genes governing mouse self-recognition by pheromones are associated with the Ir-1 locus, within the complex locus for H-2 antigens” [1]. This idea gained support by the demonstration of selective mating between congenic lines of mice differing only in segments of chromosome 17 containing the major histocompatibility complex (MHC). On the basis of these findings the occurrence of MHC-dependent olfactory cues was postulated that enable mice to recognize one another as individuals [2]. Subsequently, rewarded training experiments in a Y maze showed that mice could learn to discriminate the scent of MHC-congenic strains [3] or their urine [4]. It appeared that the MHC-encoded components critical for this recognition were the class I molecules because mice could differentiate between urine volatiles of wild type and either MHC class I mutants [5] or knockout strains deficient in the ß2m gene and therefore lacking the expression of the MHC class I heterodimers on the cell surface [6, reviewed in Refs. 7, 8]. Importantly, female mice, in which the vomeronasal organ had been surgically removed, could be trained to differentiate between urine volatiles of two MHC-congenic strains to the same extent and at the same rate as sham-operated mice, demonstrating that learning was achieved via recognition by the main olfactory organ [9].

In another approach, “habituation-dishabituation” experiments showed that rats and mice can discriminate urine from animals differing in MHC genes without training [10]–[13]. In these studies the mice could sniff at the urine and, therefore, either the main or the accessory olfactory organ or both could be involved in the recognition of MHC-dependent differences and the critical components could be volatile or non-volatile [14]. Recently, non-volatile peptides known to be MHC class I ligands were shown not only to bind to neurons in the main olfactory and vomeronasal organs but also, when added to urine, to confer specific behavioral responses [14]–[16]. While these findings assign a novel function to MHC-specific peptides in the recognition of individuality in mice, they do not preclude a role of other molecules such as the urinary volatiles [15]. The present study specifically addresses the problem of the MHC-dependence of volatile components.

Apart from training experiments, distinct activation patterns observed by sensor arrays [17], by *c-fos* mRNA expression in the main olfactory bulb of female mice [18]) and by functional magnetic resonance imaging [19] suggest that there must be differences in the male urinary volatile composition even for closely related mouse strains. However, the identification of these differences has been difficult. In an early study, Schwende et al. [20] concluded that while there seemed to be no specific volatile products that could be related to genes in the MHC, significant quantitative differences in several secondary metabolites could be observed in females of different MHC haplotypes. Singer et al. [21] and Willse et al. [22] compared the profile of components which could be extracted with diethyl ether from urine of males of two congenic mouse strains, and, while confirming the absence of qualitative differences, found that 9 of 32 and up to 80 of 370 components, respectively, differed significantly in abundance, including a variety of aliphatic, aromatic and heterocyclic components as well as two putative pheromones. By comparing the urinary volatiles from strains sharing the same background but differing in the MHC with strains having the same MHC but differing in background or with H-2 heterozygotes two studies concluded that both MHC and background genes substantially influence the volatile profile [23], [24]. Finally, Novotny et al. [25] recently suggested that MHC genes influence the amounts of testosteron-dependent pheromones, sulfur-containing compounds, and several carbonyl metabolites. Thus, while these studies agree that there are no MHC-dependent qualitative differences, there is no consensus concerning the type, number and abundance of components which differ quantitatively.

Recently, we characterized volatile components released from the body of C57BL/6 mice or from their urine [26]. In the present study, we compared the composition of urinary volatiles between inbred strains, sub-strains of an inbred strain and a mutant lacking functional MHC class I molecules. We find that strain differences in component abundance can be correlated to the provenance and genetic distance of the mice rather than the MHC. The consequences of these observations for the elucidation of the physical basis of MHC-dependent discrimination observed in several experimental models is discussed.

## Materials and Methods

### Animals

C57BL/6J mice (stock # 000664) and mice with a targeted mutation in the ß2-microglobulin gene (B6.129P2-*B2m^tm1Unc^*J, stock # 002087) were obtained from the Jackson Laboratories, Bar Harbor, Maine. Mice homozygous for the *B2m^tm1Unc^* mutation have little if any MHC class I protein expression on the cell surface and there are few CD8^+^ cytotoxic T-cells. The mutation was originally produced by Koller et al. [27] in strain 129. Backcrossing for 11 generations to C57BL/6J mice at the Jackson Laboratories ensures that nearly all loci are of C57BL/6J origin apart from chromosomal segments flanking the *ß2m* gene. C57BL/6J mice were also obtained from the Max-Planck-Institut für Immunbiologie, Freiburg, Germany. This colony was established under SPF conditions in spring 2000 from founder mice obtained from the Jackson Laboratories. These mice carried Pasteurella, Helicobacter and *Titrichomonas muris*. Strains C57BL/6NCrl (H-2^b^-haplotype), BALB/cCrl (H-2^d^-haplotype) and DBA/2Crl (H-2^d^-haplotype) were obtained from Charles River WIGA, Sulzfeld, Germany. Strain C57BL/6J was established in 1948 at the Jackson Laboratories and transferred to the NIH in 1951 (substrain C57BL/6N), which was in turn established at Charles River in 1974 (substrain C57BL6/NCrl; for details see the “Mouse Genetics Resource Manual” from the Jackson Laboratory). We use the following abbreviations for the six strains: B6J, ß2mJ, B6JF (F for Freiburg), B6NCrl, BALB/cCrl and DBA/2Crl.

### Collection of urine

Male mice (15 animals per strain) were obtained at an age of 8–9 weeks and then kept individually in standard cages on a synthetic diet [26]. After three weeks, urine was collected individually from 15 mice in a multi-compartment stainless-steel rack, which allowed separate sampling of urine and faeces (Dieter Wetzel, Detmold, Germany). During overnight urine collection, mice were supplied with water but no food. Samples from 2–4 collections obtained over two to three consecutive weeks were pooled for each mouse and stored at −20°C until used for the analysis of volatiles.

### Stir bar sorptive extraction (SBSE)

A 1.5 ml Eppendorf tube was supplied with 0.5 ml urine, 50 ng anisol as an internal standard, 150 mg NaCl and a freshly conditioned Gerstel-Twister (1 cm stir bar coated with 1 mm polydimethylsiloxan, Cat.-Nr. 011333-001-00, Gerstel, Mühlheim an der Ruhr, Germany). The samples were agitated on an IKA-Vibrax® (IKA Labortechnik, Staufen, Germany) for 24 h at a frequency of 1000/min. The stir bar was then removed, rinsed briefly with water, dried on a tissue and placed in a stainless steel tube suitable for a Thermal Desorption System (Perkin-Elmer ATD400). One set of 10 Twisters was used throughout this study.

### Gas chromatography/mass spectrometry (GC/MS)

Thermal desorption of solutes from the stir bars was performed within 10 min at 250°C and a helium flow rate of 55 ml/min. After focusing on a cryo-trap at −30°C the volatiles were separated on an Agilent J&W DB-5 column in a Hewlett-Packard GC interfaced to an HP 5973 Mass Selective Detector (see Ref. 26 for details). Mass spectra of the urine mixtures were recorded in the total ion mode for identification of components and for selection of substances for quantitative analysis. This was performed in the selected ion mode for different ensembles of 6 ions and an individual dwell time of 30 ms. For a given component, one or two characteristic fragments were used to automatically detect the peak and evaluate its abundance in retention time intervals of +/−0.5 min (see Table S1). Care was taken to ensure that the fragment or the fragment-ratio was unique for the respective component in the retention time interval. Nevertheless, for a minority of peaks, the spectra had to be evaluated individually.

### Statistics

The fragment intensity of all components for each spectrum was normalized to the respective intensity of the internal standard anisol (m/z of characteristic fragment 108). The mean and standard deviation for the abundance of each urine component was calculated. The data for the 69 components were evaluated for pair wise comparisons between strains by applying the two-tailed t-test at the level p = 0.05. Hence one expects to find 5% or three to four differences even if there are no differences at all. In order to get a rigorous estimate of the number of significant differences we use the Bonferroni correction with an effective significance level 0.05/69 = 0.00072.

## Results

### Experimental procedure

The mice used in this study were standardized with respect to sex, age, diet and group size. For each animal, the urine samples used for the analysis were mixtures from several collections. In a previous study [26], we absorbed volatiles flushed from urine by a stream of air on a porous polymer resin based on 2,6-diphenylene oxide (Tenax™ TA). After desorption by heating, components were subjected to gas chromatography/mass spectrometry (GC/MS). This technique suffers from the fact that strongly adsorbed, semi-volatiles cannot be readily thermally desorbed [28], [29] and that it is not suitable for the analysis of large numbers of samples. Therefore, we adopted the technique of sorption, which is based on the partitioning of hydrophobic components between the aqueous phase and a polydimethylsiloxane phase coated on a stir bar [28], [29]. Figure S1 demonstrates for representative solutes that an equilibrium between the aqueous phase and the polymer was reached within 24 h. The optimized procedure (see Methods for details) allowed us to estimate components in the same sample with a standard deviation of 16% (averaged standard deviations for all components of reference samples in Tables S2 and S3).

### Choice of mouse strains

We evaluated and compared the urinary volatile profile from six strains of mice (see Methods for details). The possible dependence on MHC class I-molecules was investigated by comparing C57BL/6J mice and a strain with a targeted mutation in the ß2m gene. This mutation causes deficient surface expression of MHC class I heterodimers although the larger α chains encoded in the MHC genetic locus are still synthesized (abbreviated B6J and ß2mJ, respectively). In order to study the influence of breeding conditions and genetic drift, we included two substrains of C57BL/6J (designated B6F and B6NCrl). These four strains carry the H2^b^ haplotype. Finally, two unrelated inbred strains, BALB/c and DBA/2 (designated BALB/cCrl and DBA2/2Crl, respectively) were investigated, which share the H2^d^-haplotype.

### Qualitative comparison of urine volatiles

**Figure 1:**
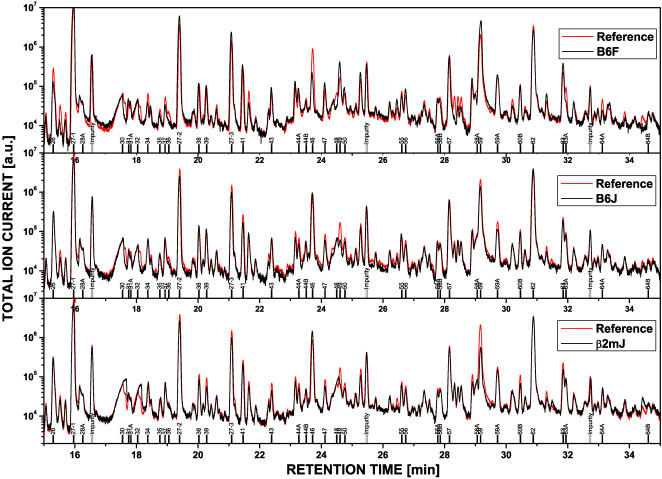
Comparison of GC/MS spectra from mouse urinary volatiles. A total of 45 urine samples were collected from B6J, B6F and ß2mJ mice (15 animals per strain). The reference spectrum (red color) is an average from three spectra obtained for mixtures of equal aliquots of 15 urine samples from 5 animals per strain (animals #1–5, 6–10 and 11–15, respectively). Spectra obtained for mixtures of equal aliquots of the 15 urine samples from each strain (black color) were aligned to the reference spectrum. The figure shows a section of the entire chromatogram plotting the total ion current on a logarithmic scale against the retention time. The peaks evaluated in the quantitative analysis are marked (compare Table S1).

Figure 1 presents sections from GC/MS spectra obtained for urine mixtures from 15 animals per strain for B6J (middle panel), ß2mJ (lower panel) and B6F mice (upper panel). Each spectrum was aligned to a reference spectrum representing the average of components detected in urine samples from all 45 mice. Comparisons of B6J, ß2mJ and B6F with the reference spectrum showed an excellent qualitative agreement in peak number and shape, i.e. there appeared to be no components that were characteristic for any of the three strains. Consequently, pair wise comparisons of the spectra for strains B6F, B6J and ß2mJ likewise revealed no obvious qualitative differences. Similar agreement was observed for components present in the rest of the chromatograms (not shown). We concluded that the three mouse strains showed no detectable qualitative differences in a total of about 180, mostly minor peaks detectable in the chromatograms.

We evaluated the GC/MS spectra for the presence of clearly defined peaks, which corresponded to 69 components (see Fig. 1 and Table S1). The majority (42 from 69) of the components have previously been detected [26]. Furthermore, most new components (18 from 27) eluted at late retention times suggesting that these were readily desorbed from polydimethylsiloxan stir bars but not from Tenax™ TA and hence had failed detection in the previous study. Therefore, the spectrum of components obtained with coated stir bars reflected the composition of urine scent as well or better than that acquired by the purge and trap procedure using Tenax™ TA. For example, the components commonly considered to be mouse pheromones (6-hydroxy-6-methyl-3-heptanone, 2-heptanone, 2,3-dehydro-exo-brevicomin, 2-*sec*-butyl-4,5-dihydrothiazole and ß-farnesen) were reproducibly detected by both procedures.

**Figure 2:**
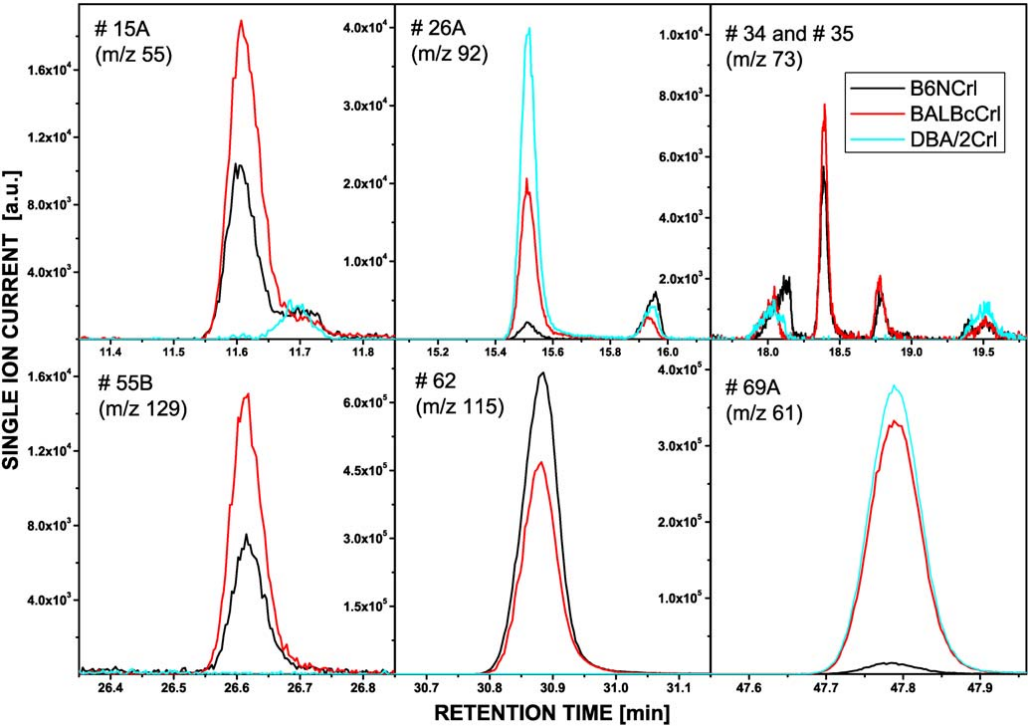
Abundance of selected urine components in B6NCrl, BALB/cCrl and DBA/2Crl mice. Total ion current GC/MS spectra were obtained for urine mixtures (15 animals per strain) in duplicate. The spectra were aligned and inspected for peaks showing large differences. Six such peaks are depicted by the relative intensity of their characteristic fragment ions (average from two spectra) against the retention time. Component # 62 refers to 2-*sec*-butyl-4,5-dihydrothiazole, the structure of the other components is unknown. Their characteristic fragments (relative intensity in brackets) are #15A [39 (8), 40 (6), 41 (8), 52 (5), 54 (51), 55 (100)], #26A [45 (100), 46 (25), 47 (35), 48 (14), 61 (35), 76 (11), 77 (24), 92 (87), 94 (39)], #34 [39 (51), 41 (61), 54 (63), 55 (100), 56 (41), 57 (29), 59 (30), 73 (43), 86 (16)], #35 [39 (43), 41 (58), 43 (23), 54 (60), 55 (100), 56 (37), 57 (33), 59 (27), 73 (38), #55b [39 (6), 41 (15), 43 (12), 45 (13), 59 (27), 60 (100), 61 (7), 114 (16), 129 (27)] and #69A [35 (6), 45 (19), 46 (8), 47 (5), 61 (100), 63 (5), 93 (5), 186 (13)].

While we noted no qualitative differences between strains B6J, ß2mJ and B6F), we observed qualitative or large quantitative differences when we compared B6NCrl, BALB/cCrl and DBA/2Crl mice (Fig. 2). Five components (GC/MS peaks # 15A, 34, 35, 55B and 62) were present in both B6NCrl and BALB/cCrl mice but absent in DBA/2Crl mice. Interestingly, DBA/2Crl mice lacked 2-*sec*-butyl-4,5-dihydrothiazole (peak #62), which is a candidate pheromone [30]. Conversely, components #26A and 69A were present in very low concentrations in B6Crl mice but clearly detectable in the other two strains.

### Quantitative comparison of urine volatiles

We analyzed the selected 69 peaks for a quantitative comparison of all six strains. From the total ion mass spectrum of each peak we chose one or in some cases two characteristic fragments (cf. Table S1) and then recorded the GC/MS spectra in the selected ion mode. This procedure improved the sensitivity of peak detection, reduced the background of the spectra and allowed their automatic evaluation. The results of a total of 50 spectra (15 urine samples each for B6J, ß2mJ and B6F mice and five controls from a urine mixture) are presented in Table S2. In an independent experiment the same number of spectra were obtained for samples from B6NCrl, BALB/cCrl and DBA/2Crl mice (see Table S3).

As noted previously [26], for some components the abundance showed a very high, for others low variability within the groups of mice considered. Overall, the standard deviation of the mean for 15 measurements varied between 18 and 308% and thus for essentially all components was much higher than the mean standard deviation in the reference samples (16%). This variability implied that the odor bouquet differed even for individuals of the same strain. This conclusion was supported by the analysis of individual urine samples collected over 3 weeks from five B6NCrl mice (cf. Fig. S2). Comparison of these animals showed that many components occurred at closely the same relative abundance while others varied up to 10-fold. Overall, within the time frame investigated the individual odor differences were relatively stable.

The similarity (or distance) of the mouse strains with respect to the individual volatile concentration was evaluated by two-tailed t-tests for the means with Bonferroni correction for 69 components at p = 0.05. This procedure yielded for each pair wise comparison a set of components with significantly different concentration. Probably not all of these significant differences can be detected by a mouse. For example, as shown for octanol as a model compound, a 3-fold difference is just sufficient to be discriminated by mice [31]. Therefore, we have computed for each significant compound the quotient, r, of the larger and the smaller mean, such that r>1 (see Table 1). Table S1 lists the r values for the pair wise comparisons between strain B6J and the other five strains and indicates the distribution of significant differences.

**Table 1:** Relatedness of mouse strains by comparison of urinary volatile abundance.

		β2mJ	B6F	B6NCrl	BALB/cCrl	DBA/2Crl
r>1	B6J	5	19	22	25	33
	β2mJ	–	23	26	23	34
	B6F		–	21	35	28
	B6NCrl			–	12	16
	BALB/cCrl				–	11
r>2	B6J	2	12	22	23	32
	β2mJ	–	16	25	21	31
	B6F		–	20	34	25
	B6NCrl			–	10	16
	BALB/cCrl				–	11
r>3	B6J	0	3	9	12	25
	β2mJ	–	4	15	17	27
	B6F		–	12	21	18
	B6NCrl			–	4	14
	BALB/cCrl				–	8
r>5	B6J	0	2	5	6	14
	β2mJ	–	2	4	10	16
	B6F		–	7	12	15
	B6NCrl			–	2	8
	BALB/cCrl				–	6
r>10	B6J	0	1	3	3	9
	β2mJ	–	1	2	5	8
	B6F		–	4	9	12
	B6NCrl			–	0	5
	BALB/cCrl				–	6
r>100	B6J	0	0	0	0	3
	β2mJ	–	0	0	0	2
	B6F		–	0	0	2
	B6NCrl			–	0	4
	BALB/cCrl				–	3

The number of significantly different components in the ensemble of 69 urinary volatiles was calculated for all strain combinations. The ratio, r, was obtained by computing for each significant compound the quotient, r, of the larger and the smaller mean, such that r>1. The total number of significant differences are listed in the upper section of the table, i.e. the means of these components differ by a factor, r>1. The lower sections list the fractions of these components, which differ by more than 2-fold, 3-fold, 5-fold, 10-fold and 100-fold, respectively.

The urine of the B6J and ß2mJ mice differed significantly in 5 of 69 components (7%), the lowest value observed in the five comparisons. Surprisingly, there were 19 (28%) and 22 (32%) components different between strains B6J vs. B6F and B6J vs. B6NCrl, respectively. Differences increased to 25 (36%) and 33 (48%) components, respectively, when B6J mice were compared to BALB/6Crl and DBA/2Crl mice. Therefore, in comparison to strain B6J the fraction of differences increased in the order ß2mJ<<B6F<B6NCrl<BALB/cCrl<DBA/2Crl (cf. upper section of Table 1 for ratios r>1).

Strains B6J and ß2mJ differed in 5 components, but only 2 thereof (r = 2.25 for component #11 and r = 2.14 for #39) differed by a factor more than 2-fold and none by more than 3-fold. Furthermore, these two compounds also differed between B6J and either B6NCrl (#39) or both B6F and B6NCrl (#11) indicating that these differences did not correlate to the ß2m mutation. In contrast, strains B6J as well as ß2mJ showed numerous components differing more than three- and some even 10-fold when compared to strains B6F or B6NCrl. Therefore, strains B6J and ß2mJ were much more similar to each other than to the B6-substrains, B6F and B6NCrl. As may be expected, a much larger number of components differing by higher r-values (from >3 to >100) was observed when the urine odor of the three inbred strains B6, BALB/c and DBA/2 was compared. In summary, evaluation of the magnitude of differences also showed the close similarity of the B6J wild type and its mutant as well as the increasing dissimilarity, when these strains are compared to the two B6 substrains or to the two other inbred strains.

## Discussion

This study demonstrates that standardization of urinary volatile collection and analysis as well as an adequate sample size allows the reproducible detection of an ensemble of components. As can be expected, inbred mouse strains separated by nearly a century [32] exhibit some qualitative or large quantitative differences. Of particular interest is the lack of 2-*sec*-butyl-4,5-dihydrothiazole in DBA/2 mice, which has been implicated as a pheromone in estrus induction, puberty acceleration, female attraction and inter-male aggression [30]. This component may alter the behavior of this mouse strain when added to urine.

The abundance of the urinary components can be discussed on two levels. First, some components vary widely between individuals from the same strain, implying that it is unlikely that two urine samples will have exactly the same scent. In addition, a large individual variability in the amount of certain volatiles was also observed in mouse body odor [26]. It is therefore possible that these differences enable a mouse to identify individuals of an inbred strain by the urine or body odor or both, analogues to the differentiation of the scent of monozygotic twins by bloodhounds [33]. Second, it appears that the fraction and extent of quantitative differences (Table 1) between urinary components reflects the relationship between the mouse strains with regard to genetic distance and environmental factors such as the commensal flora or the diet used by the suppliers for raising the animals before their delivery to the Tübingen laboratory. B6J and ß2mJ mice were obtained from the Jackson Laboratories, which enforce rigid breeding programs to limit genetic drift. Nevertheless, 7% of the components were found to be significantly different in abundance, albeit at most by 2-3-fold. B6F and B6NCrl are sub-strains of B6J obtained from two other sources. The fraction of differences and the extent of these differences in comparison to strains B6J and ß2mJ strongly increased. As may be expected, differences increased even further when these strains were compared to BALB/cCrl and DBA/2Crl. The importance of environmental factors can be inferred from the observation that in comparison to strain DBA/2Crl, strains B6NCrl and BALB/cCrl mice, i.e. animals provided by the same supplier, show fewer significant differences, 23% and 16%, respectively, than B6J (48%) and B6F mice (41%, cf. Table 1). Therefore, quantitative differences appear to be due to subtle phenotypic and genotypic changes in the complex physiology of the mouse strains, which have been separated by inbreeding for many generations (compare Ref. 12 and the literature cited therein for divergence by random mutations and various phenotypic differences documented for congenic strains). All urinary components analyzed here are components produced by so far uncharacterized, minor metabolic pathways, which appear to be under relatively loose control causing considerable within- and between strain variability in abundance. In a comparison of two congenic C57BL/6-lines (haplotypes H-2^b^ and H-2^k^) previous studies have reported quantitative differences in a different set of urinary components using a preparation technique that was biased towards the detection of less volatile solutes [21], [22]. Therefore, variability in abundance appears to be characteristic for a large fraction of all urinary volatiles.

What are the consequences of these results for the concept that the MHC directly or indirectly influences the odor composition of mouse urine and is therefore important for recognition of individuality in mice [1]–[4]? Considering the numerous quantitative differences observed for B6 sub-strains, we are reluctant to assign the few and small differences between B6J and ß2mJ mice to an influence of functional MHC class I expression. For the same reason, we would argue that differences in the quantity of urinary volatiles found between two C57BL/6-congenic lines, which were established decades ago and have since been propagated separately, cannot be interpreted readily as being dependent on genes in the MHC [21], [22]. The hypothesis that genes governing mouse-self recognition by pheromones are associated with the MHC [1] was formulated at a time when only a few genes of this complex were known. Sequencing of the MHC has as yet not uncovered candidate genes for regulating the abundance of secondary metabolites.

How can our results be reconciled with studies invoking an influence of the MHC on urinary volatile composition? We propose that the quantitative differences in the bouquet of volatiles between closely related strains result in distinct activation patterns of *c-fos* mRNA expression in the main olfactory bulb [18] as well as of sensor arrays [17]. At present, these studies have not been directly related to analytical data on urine scent. In the Y-maze experiments, mice were trained to differentiate urinary volatiles from MHC-disparate donors (reviewed in Refs. 7 & 8). Because these studies used urine samples from many individuals (see e.g. Ref. 4), the sensor mice must have learned to recognize the MHC-specific difference (or a combination of multiple differences) against the variable volatile profile from these individuals. This difference was then treated as an MHC-specific trait that could be successfully inherited to F_2_-segregants of two H-2 congenic strains [4], [34], of *H-2^b^* wild type and *H-2^bm1^* mutant mice [5] or of B6 and ß2m-deficient mice [6]. These segregation experiments provide the strongest argument for a specific influence of the MHC on the odor profile and, in principle, a similar segregation experiment could be performed for the odor profiles. However, the small differences in the abundance of urinary volatiles observed between B6J and ß2mJ mice (Table 1) cannot be considered to be robust genetic traits. Therefore, we do not expect that an extension of the present investigation to F_2_-segregants would yield results which could be interpreted in terms of an MHC association. Since we have quantitatively analyzed only a fraction of the total number of volatiles present in the urine, one can argue that we have missed the critical components, which are controlled by and consequently would segregate with the MHC. While this remains a possibility, it should be noted that the agreement between the aligned GC/MS spectra between B6J and ß2mJ mice (Fig. 1) is good even in regions not selected for the quantitative analysis. In summary, the behavioral experiments [6] and the compositional data can only be reconciled by postulating as yet undetected MHC-dependent differences in urinary trace volatiles, which a mouse can learn to differentiate after extensive training. It can be predicted that it would be very difficult to train mice to differentiate volatiles from urine mixtures, e.g. from groups of B6J vs. ß2mJ mice compared to B6J (group1) vs. B6J (group 2) and ß2mJ (group1) vs. ß2m (group2), as used in this paper (Fig. 1). The importance of using urine mixtures rather than samples from individual animals in behavioral experiments has been noted previously [11], [12].

The MHC has been proposed to influence the odor composition in humans [35]–[37]. In recent studies, Curran et al. [38] and Penn et al. [39] compared volatile organic compounds present in armpit sweat samples from different individuals. As can be expected from an outbred population, the spectrum of volatiles varied widely in both quality and quantity. Based on the analysis of close to 400 components, each individual appeared to have a unique odor profile [39]. Therefore, in humans as well as in mice the individual odor bouquet is a function of complex ontogenetic, genetic and environmental factors and a specific influence by the MHC remains to be defined.

## Supporting Information

Figure S1 Kinetics of partitioning of urine components into polydimethylsiloxane-coated stir bars. Mouse urine (6 ml) was saturated with NaCl (1.8 g) and duplicate samples (0.5 ml) were agitated with stir bars for the indicated times. The average signal intensity of the total ion current is normalized and is plotted against the equilibration time.
(0.73 MB TIF)

Figure S2 Urinary volatile profile of mice as a function of time. Urine samples from 5 B6NCrl mice taken every seventh day over 3 weeks were analysed for a selection of urinary components specified in Table 2 of the Supplementary Material. Four spectra for each mouse were evaluated, averaged and normalized to the mouse with the lowest mean for the respective component. Horizontal bars indicate the standard deviation of the mean, which is indicated by a square. The color code corresponds to volatile abundance for each of the 5 animals.
(1.36 MB TIF)

Table S1 Comparison of the abundance of urinary components between strain B6J and the other strains. Substance number refers to components previously reported (without capital letters) or newly analyzed in this study (with capital letters). The retention times and the characteristic ion fragments used in the calculation of the peak area are indicated. Component # 27 is the summed intensity from peaks at retention times 15.93, 19.37 and 21.06 min. Components of unknown structure are defined by the mass and relative abundance (in brackets) of characteristic fragments. The numbers on right side refer to the ratio of the means between strain B6J and each of the five other strains. For a given strain X, this ratio was calculated by dividing B6J/X or X/B6J for means B6J>X or X>B6J, respectively. Therefore, these ratios are always >1. Significant differences are shaded and enumerated at the bottom.
(0.06 MB XLS)

Table S2 Abundance of urinary volatiles of B6J, β2m and B6F mice determined by GC/MS in the single ion mode after normalization to the intensity for the anisol standard. The individual mice are numbered. Reference refers to five inter-dispersed measurements of a mixture of equal volumes of urine from B6J01-05, B6F01-05 and β2mJ01-05 mice. The results of the two-tailed t-test (TTEST) for the means with Bonferroni correction for 69 components at p = 0.05 are listed at the bottom. Significant differences are shaded.
(0.10 MB XLS)

Table S3 Abundance of volatile components from urine of B6NCrl, BALB/cCrl and DBA/2Crl mice. The reference refers to a mixture of equal amounts of urine from five urine samples from different mice for each strain. The occurrence of the fragment m/z 60 used for the quantification of 2-sec-butyl-4,5-dihydrothiazole (component #62) suggested its presence of in several of the samples from DBA/2Crl mice. Repetitions of these analyses with fresh urine samples from the same mice were completely negative in accordance with results from urine mixtures (cf. Fig. 2). Therefore, we concluded that urine from DBA/2Crl mice contained less than 0.1% of component #62 in comparison to the two other strains. For t-tests compare Table S2.
(0.10 MB XLS)
