# Supplementary figures and images for: Quantitative Analysis of Mouse Urine Volatiles: In Search of MHC-Dependent Differences
Source: PLoS One. 2007 May 9;2(5):e429. doi: 10.1371/journal.pone.0000429 (PMC1855987; doi:10.1371/journal.pone.0000429)

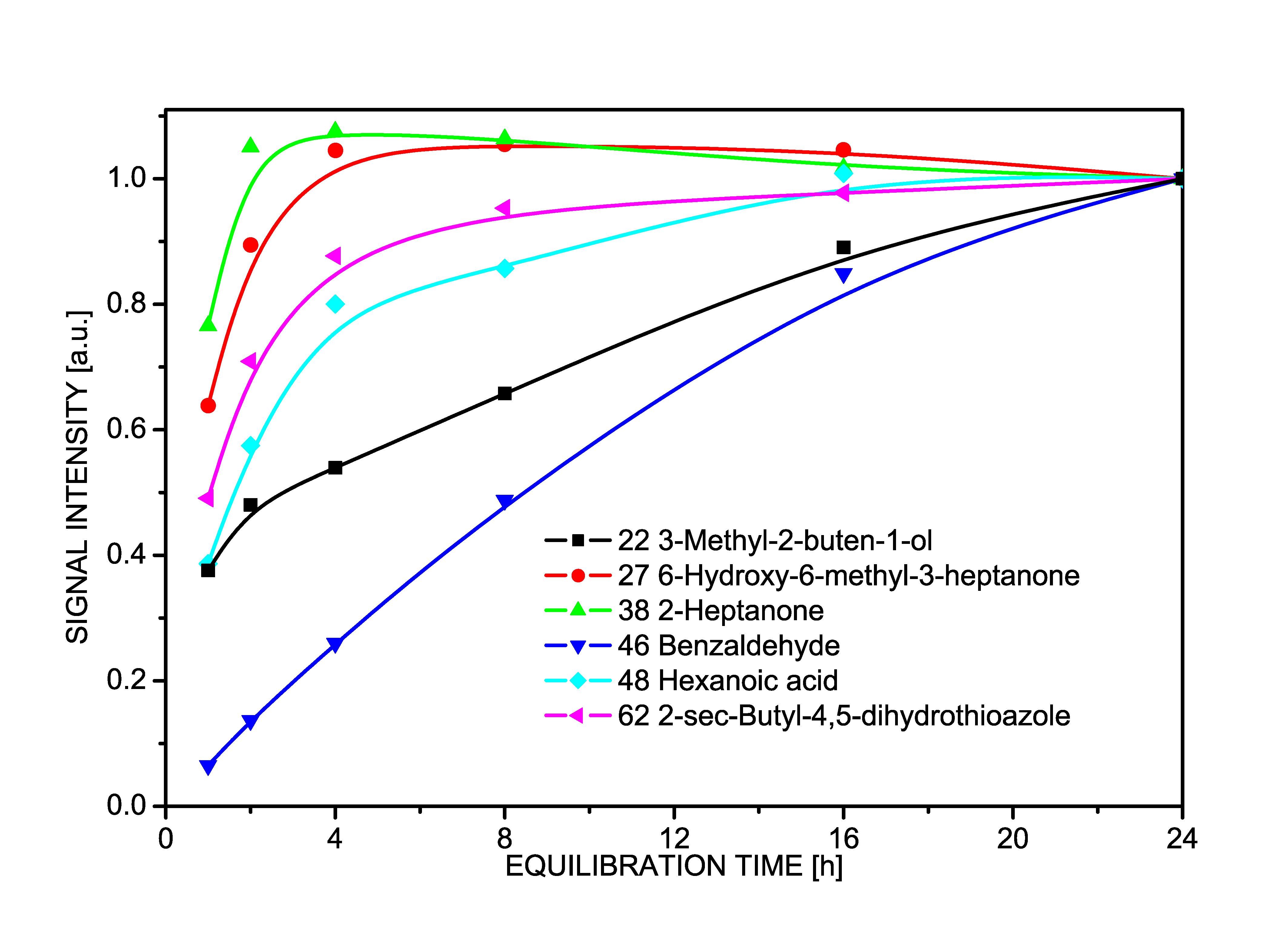

Supplement: Figure S1 — Kinetics of partitioning of urine components into polydimethylsiloxane-coated stir bars. Mouse urine (6 ml) was saturated with NaCl (1.8 g) and duplicate samples (0.5 ml) were agitated with stir bars for the indicated times. The average signal intensity of the total ion current is normalized and is plotted against the equilibration time. (0.73 MB TIF) [file pone.0000429.s001.tif]

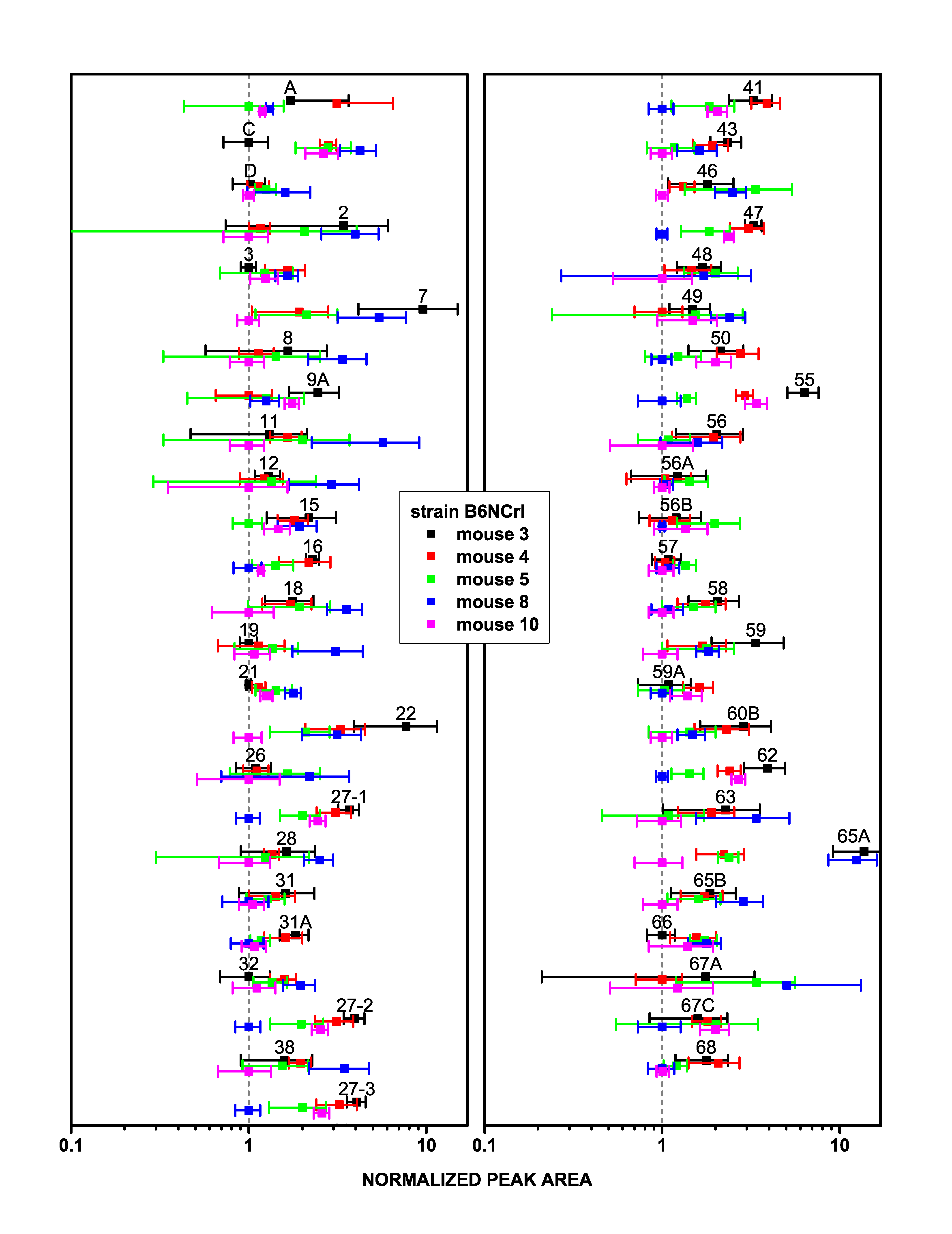

Supplement: Figure S2 — Urinary volatile profile of mice as a function of time. Urine samples from 5 B6NCrl mice taken every seventh day over 3 weeks were analysed for a selection of urinary components specified in Table 2 of the Supplementary Material. Four spectra for each mouse were evaluated, averaged and normalized to the mouse with the lowest mean for the respective component. Horizontal bars indicate the standard deviation of the mean, which is indicated by a square. The color code corresponds to volatile abundance for each of the 5 animals. (1.36 MB TIF) [file pone.0000429.s002.tif]
